# A Multi-Directional Pixel-Swapping Approach (*MPSA*) for Entropy-Retained Reversible Data Hiding in Encrypted Images

**DOI:** 10.3390/e25040563

**Published:** 2023-03-25

**Authors:** Shaiju Panchikkil, V. M. Manikandan, Yudong Zhang, Shuihua Wang

**Affiliations:** 1 Department of Computer Science and Engineering, SRM University-AP, Amaravati 522502, Andhra Pradesh, India; 2 School of Computing and Mathematical Sciences, University of Leicester, University Road, Leicester LE1 7RH, UK

**Keywords:** reversible data hiding, entropy, secure data transmission, block partition, pixel swapping

## Abstract

Reversible data hiding (RDH), a promising data-hiding technique, is widely examined in domains such as medical image transmission, satellite image transmission, crime investigation, cloud computing, etc. None of the existing RDH schemes addresses a solution from a real-time aspect. A good compromise between the information embedding rate and computational time makes the scheme suitable for real-time applications. As a solution, we propose a novel RDH scheme that recovers the original image by retaining its quality and extracting the hidden data. Here, the cover image gets encrypted using a stream cipher and is partitioned into non-overlapping blocks. Secret information is inserted into the encrypted blocks of the cover image via a controlled local pixel-swapping approach to achieve a comparatively good payload. The new scheme MPSA allows the data hider to hide two bits in every encrypted block. The existing reversible data-hiding schemes modify the encrypted image pixels leading to a compromise in image security. However, the proposed work complements the support of encrypted image security by maintaining the same entropy of the encrypted image in spite of hiding the data. Experimental results illustrate the competency of the proposed work accounting for various parameters, including embedding rate and computational time.

## 1. Introduction

The communication network has been growing at a rapid pace, and so has content sharing. Furthermore, the number of internet users has grown substantially. A secure way of communication plays a vital role in shielding secret information. Hence, cryptography and data-hiding approaches have been in focus [1,2]. Cryptography methods manipulate data, such that the modified form of data could ensure the confidentiality of the content during transmission over an unsecured network. The encrypted data will have a high amount of randomness, and anyone can distinguish the original data from the encrypted data. The attackers may think that the encrypted data consist of some crucial information and will try out cryptanalysis to decode the actual data. Data hiding came as an alternative to cryptography in which we hide the data within a cover medium. The cover medium can be audio, video, image, or text file. The digital content resulting from the data-hiding process looks similar to the original cover content, and the attackers will be completely concealed from the knowledge of the hidden information. So the possibility of decoding attempts on such content will be lower compared to the cryptanalysis of the encrypted data.

**Figure 1:**
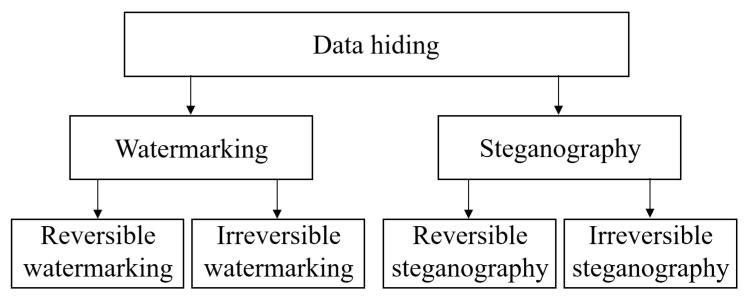
Data-hiding approaches.

Figure 1 shows the types of data-hiding approaches.

1. Digital watermarking: In digital watermarking, unique information gets embedded within the cover medium, which will be used for data authentication or copyright protection [3]. Unique information, for example, a logo image or a unique bit sequence, forms a watermark. The embedded watermark gets extracted for data authentication or copyright protection. For this purpose, the original watermark gets compared with the extracted watermark. Robust watermarking schemes or fragile watermarking schemes are employed depending on the application. For copyright protection, the preferred scheme is a robust watermarking scheme since the embedded watermark will not be destroyed by common image processing operations such as compression, scaling, rotation, etc. [4]. Fragile watermarking schemes are useful for data authentication in which the embedded watermark cannot survive any kind of operation on the watermarked image [5]. So any unauthorized operations in the watermarked image can be claimed by extracting the hidden watermark. Reversible watermarking is different from irreversible watermarking. In reversible watermarking, apart from extracting the embedded watermark, the cover medium should also be recovered.
2. Steganography: Steganography is similar to the watermarking approach that varies in its purpose. In steganography, the information hidden is much larger in capacity, and the receiver does not care about the cover image used for concealing purposes. Here, the cover medium at the receiver end is not so significant after the extraction of hidden information [6]. As noted in the reversible watermarking, the cover medium is significant in reversible steganography.

**Figure 2:**
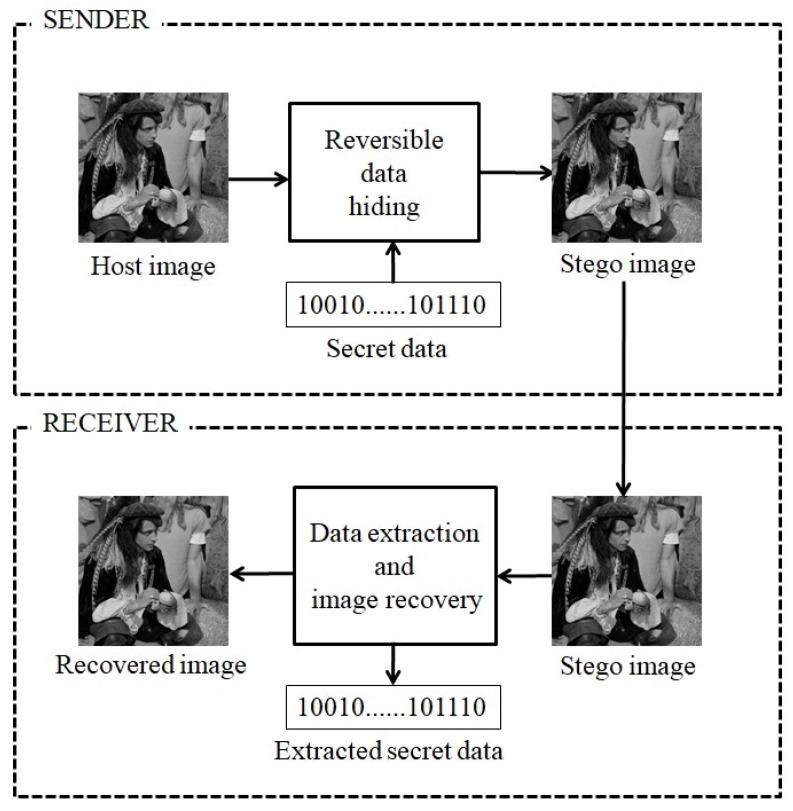
Flow diagram of reversible data-hiding scheme.

Reversible data-hiding (RDH) schemes are derived from steganography, which aims at lossless recovery of the actual cover medium, along with the extraction of the hidden secret information [7,8]. In this technique, the data are embedded in mediums such as an image, videos, or audio. Apart from this, a hologram is another example of the cover medium used in RDH [9,10]. The crust of RDH is in extracting the embedded data and recovering the actual cover medium, both in a lossless manner [11,12,13]. Hence, both the medium and the data are significant. A few applicable areas of RDH are medical image transmission, satellite image transmission, defense intelligence operations, and criminal investigations. The basic working of RDH is given in Figure 2.

Two main approaches in RDH are histogram shifting and difference expansion. In the case of difference expansion-based RDH, the adjacent pairs of pixels are selected, and their difference is expanded to hide the message bits [14,15]. Overflow and underflow are the two major concerns with difference expansion-based RDH schemes. In comparison, one can identify the highest frequency of intensities via a histogram of the image, and data are embedded relying on this maximum value in the histogram [16,17,18]. This method is termed the histogram shifting-based approach that gives a high payload depending on the highest frequency of intensities in the histogram.

**Figure 3:**
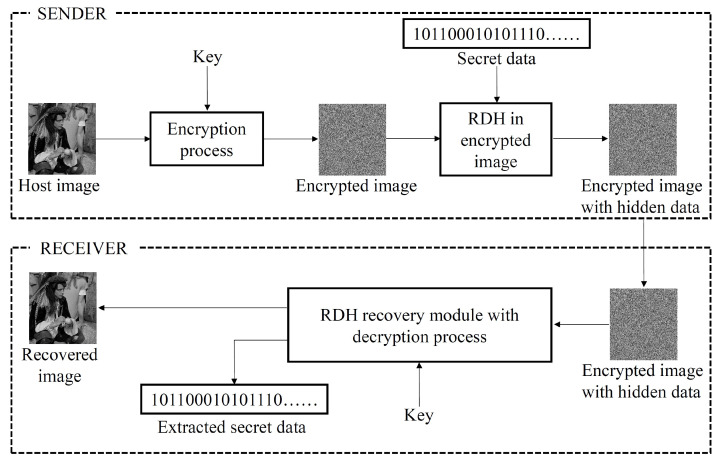
Flow diagram of reversible data-hiding scheme in encrypted image.

RDH in natural images [19] and RDH in encrypted images [20] are the two widely explored types of RDH. A basic workflow of RDH in an encrypted image is shown in Figure 3. In an encrypted domain, data can be reversibly embedded either over an encrypted image or through encryption. In the first case, two parties are involved at the sender’s end. They are the content owner and the data hider. Here, image encryption is performed by the content owner, and hiding data becomes the responsibility of the data hider. In this scenario, the details of the original image are concealed from the data hider, while in data hiding through encryption, both the encryption and data-hiding processes are handled by a single party.

The proposed MPSA scheme is an RDH scheme in encrypted images. The contributions that are specific to the proposed scheme are as follows:

1. Retainment of entropy and histogram: The bulk of the existing RDH schemes in encrypted images predominantly alters the pixel distributions in the encrypted image during data hiding. However, this manuscript proposes an RDH scheme called the MPSA scheme that retains the entropy and histogram. It is noteworthy to mention that entropy and histogram are the two efficiency parameters to judge the efficiency of any image encryption process. The proposed scheme is well poised with entropy and histogram retainment even after data hiding and, hence, does not alter the efficiency of the image encryption process.
2. Embedding capacity is independent of the structural properties of the cover image: The MPSA scheme is completely independent of the structural properties of the cover image. This ensures a fixed embedding rate by the proposed MPSA scheme.
3. Real-time performance: The practical computation time incurred during the embedding and extraction phase is much less, proving its real-time applicability.
4. Less overhead at the receiver side: Machine learning models are explored in RDH to improve the efficiency of the recovery process. However, these trained models must be shared with the receiver before the image transmission. In comparison, the MPSA scheme introduced in this paper does not have such overheads since it is based on some statistical measures.

Furthermore, the manuscript discusses literature works on RDH schemes in the encrypted images in Section 2, the MPSA approach in Section 3, an illustration of the work with an example for better understanding in Section 4, and the results obtained from the proposed work, with a detailed comparative study in Section 5. The manuscript is concluded in Section 6 with a few insights into future works.

## 2. Related Work

In this section, we discuss a few RDH schemes in encrypted and non-encrypted images. The scheme reported in [21] proposed by Zhang in 2011 is the first universally accepted RDH scheme in encrypted images. The scheme is widely used by researchers as a reference work and improved in later years. Here, the secret data are embedded in the three least significant bits (LSB) of the encrypted image. Initially, the image is divided into non-overlapping blocks, with each block being classified into two parts. To embed a bit 1, the last three LSBs of one part of the block were flipped, and the other part of the block was used to embed a bit 0. An improved version of the above work was proposed in [22]. The smoothness measurement used for block recovery was improved, and used a side-match technique to recover the embedded secret data. The vertical absolute difference and the horizontal absolute differences of pixels in a block were considered to calculate the smoothness to bring a significant improvement in block recovery.

The RDH scheme proposed in [23] uses a histogram-based approach, where the data-embedding capacity was improved. The histogram of the encrypted image was generated, and the peak value was found. All those pixels whose intensity was the same and higher as compared to that of the pixel which was identified for peak value are incremented to create room for embedding the secret information, whereas two methods called joint method and separable methods were discussed in [24]. In the joint method at the receiver end, an improved adaptive interpolation algorithm was introduced to quantify the relation existing among a pixel and neighborhood pixels. In this method, both the process of extracting data and image recovery are interlinked. In the second method, both these processes were handled separately. The pixel for embedding data is selected in such a way that its neighboring pixels are not altered. In the separable method, the most significant bits of the selected bit are modified to embed data. Hence, a median filter is used to overcome the noise introduced. Compression with encrypted images and data embedded on the compressed portion was introduced by [25]. The encrypted image was classified into the chessboard fashion, where the white locations were selected for compression and data embedding. Half of the fourth LSB was identified to embed the secret data.

A bit of secret data is embedded in every block using the data-hiding key [26], but in comparison to earlier schemes, while encrypting the image, all except the fourth bit of each pixel gets encrypted. This fourth bit is hence used to embed the data. Using the smoothness characteristics along the isophote direction, the secret bits are extracted. A reduction in the extracted bit error rate considering the appropriate block size was proposed in [27]. Here, a block complexity function is defined to facilitate the correct recovery of the block. When compared to other schemes, the embedding data ratio is also defined to embed the secret data. A progressive recovery-based RDH technique was proposed by [28], wherein three channels of the encrypted image were made to embed different amounts of secret bits, unlike any other scheme. They used pseudo-random permutation to create three channels of encrypted image pixels. Each pixel in different channels is later compressed to make room for embedding the secret data.

Instead of creating different channels as in [28], the encrypted image is partitioned into four equal groups and two data-embedding methods were utilized. A cyclic shifting of bits in a pixel to embed secret data in three groups and a data swapping-based embedding algorithm was used as a second round of embedding. Another method to classify the encrypted image into two by using run-length coding along with Huffman coding was introduced by [29]. Hence, the data hider has to conduct different mechanisms to embed the secret data into the classified blocks using the data-hiding key. As in many other schemes, the room-vacating approach is explored to embed secret data. Another RDH scheme which also accommodates secret data via vacating room was proposed by [30]. To mask the original image contents, block-level permutation and stream cipher encryption were used. The most significant bit (MSB) layers where the secret data could be embedded are adaptively compressed based on the frequency of MSB, thus embedding additional data. Unlike most of the existing schemes, ref. [31] reserved the data-embedding space before encrypting the original image via the prediction error estimation method. A location map is also used to justify whether the information could be embedded in a particular location. Anyway, the MSBs of selected location pixels are used for secret data hiding as many other schemes. Machine learning models are also used in RDH. In [32], a convolutional neural network (CNN) framework supports recovering the cover image and hence the embedded data from the marked image. Here, the data embedding is made possible through an image-scrambling algorithm called the Arnold transform algorithm.

Secret data can also be embedded in a non-encrypted domain. The quality of the stego image is very important as the original pixels are modified while embedding the data [33]. Unlike in the encrypted domain, CNN is also used as a predictor for predicting a set of pixels from another set in RDH in non-encrypted images [34]. The RDH scheme utilized the feature extraction potential with multiple receptive fields and the global optimization capability of the CNN model. The experimental results proved that the CNN-based predictor is more accurate in predicting the pixels over the linear predictors. RDH on non-encrypted images is also exploited in the compressed domain [35]. Here, the first level of embedding is carried out using the mean and standard deviation values obtained through the absolute moment block truncation coding (AMBTC) on the image blocks [36]. The second level embedding is attained via (7,4) hamming code on the AMBTC-based image. Histogram shifting for embedding additional information in non-smooth regions of an image introduces a higher distortion. A technique to reduce the pixel’s invalid shifting will produce a higher-quality stego image [37]. The image pixels are classified into two groups accounting for a checkerboard pattern. Fluctuations in each group are calculated and combined with prediction errors helps to achieve a higher embedding rate with information being embedded by modifying the prediction errors. A similar kind of approach is discussed in [38]. Here, an efficient embedding capacity is computed while reducing the distortions introduced with the embedding of additional data. They identify multiple peaks from regions with less local complexity from the histogram and are expanded to hide the additional information. The focus of the experiment relied upon achieving better embedding with the least distortion.

To embed the secret information, most of the existing schemes have been utilizing the vacant room created by compression or by shifting or finding pixel differences, and a few have tried modifying the original bits of the pixel, either MSBs or LSBs. A sufficient amount of redundant space is required to improve the embedding capacity. We should also reduce the complexity that leads to errors during the recovery process. Here, we propose a novel MPSA scheme that particularly retains the encrypted image pixel’s intensities as such to embed the secret information.

A detailed literature review reveals that the existing RDH schemes in encrypted images have low embedding rate, and there is wide scope to improve this parameter without compromising the image restoration capability. Another important observation is that the majority of the pre-existing works on RDH in encrypted images distort the encrypted pixel values, which may result in a new pixel distribution in the marked image. A uniform pixel distribution is expected from an encrypted image, and the unusual pixel distribution in the image due to the data-hiding process will lead to security concerns. In this research, a novel MPSA RDH scheme is disclosed with an acceptable embedding capacity that does not change the distribution of the pixel in the encrypted images during the data-hiding process.

The advantages of the proposed MPSA RDH scheme are as follows:

1. Most of the existing schemes modify the entropy of the encrypted image after hiding the additional information, while the proposed scheme does not. Thus, the proposed scheme comes under the banner of a secured RDH scheme from the encryption algorithm purview;
2. A slight change in the entropy of the fully encrypted image is an indication of the presence of information. As we retain the same entropy of the encrypted image, the proposed scheme does not expose the presence of hidden information;
3. The properties of the proposed MPSA RDH scheme are very much suitable for highly secure and sensitive applications, such as medical image communication, military applications, satellite image communication etc.

## 3. Proposed Work

The MPSA scheme is devised carefully to ensure the same entropy measure of the encrypted image and the marked encrypted image. Here, at first, we encrypt the cover image using a stream cipher. The encrypted image is handled as blocks of identical size. Each block is processed to embed the secret data. For this, a special technique called local pixel swapping is introduced. Furthermore, two bits of data are embedded within each block using the proposed scheme. It is always important to maintain the correlation of the pixels in an image, which is exploited in the proposed technique for better cover image recovery. The scheme is evident enough to retain the entropy of the encrypted image even after data embedding. The proposed work is divided into two phases: the data-hiding phase and the image-restoration phase. The first phase explains how to embed certain information on a cover image through the proposed local pixel-swapping technique, and the second phase discloses how to retrieve the cover image and hidden information from the marked image. Initially, the encrypted image *E* is fed as an input to the RDH system. The system handles the input image as blocks of identical size without overlapping.

### 3.1. Data-Hiding Phase

In this phase, data are embedded in an image *E* of size X×Y pixels. The image *E* is considered to be an encrypted image, processed as independent H×H blocks. Thus, the encrypted image *E* is given as follows:

(1) $$E=E_{H\times H}^{1},E_{H\times H}^{2},\ldots ,E_{H\times H}^{N},$$

where *N* is the maximum blocks of size H×H pixels, derived from *E*, which are not overlapping. Now,

(2) $$N=\left\lfloor \frac{X}{H}\right\rfloor \times \left\lfloor \frac{Y}{H}\right\rfloor .$$

Furthermore,

(3) $$E=\overset{N}{\underset{i=1}{⋃}}E_{H\times H}^{i}.$$

Since the newly introduced MPSA scheme allows the data hider to conceal two bits of information in one image block, the secret message *D* is chosen accordingly. The properties of the secret data *D* are mathematically defined in Equations (4) and (5).

(4) |D|=2.N.

(5) $$D=\left(X_{1},X_{2},\ldots ,X_{i}\right),\;\;\;\;\mathrm{where}\;\;X_{i}\in \left\{0,1\right\}\;\;\;\;\mathrm{and}\;\;\;1\leq i\leq 2.N.$$

We know that when the size of the encrypted image is 512×512 pixels, processed as 4×4 pixel blocks, the total blocks accounted for is then 16,384. Hence, the actual amount of data bits that we can hide with the proposed scheme is 2×16,384=32,768 bits. Algorithm 1 processes *E* as non-overlapping blocks to embed the secret information. In the current scenario, each 4×4 block is again divided into independent 2×2 blocks to hide 2 bits in a 4×4 block. The pixel values at different positions of a 2×2 block are swapped in a predefined manner, previously referred to as local pixel swapping, to embed particular 2-bit information. Algorithm 1 gives the details of the information embedding process.
**Algorithm 1:** Proposed pixel-swapping-based approach for RDH.
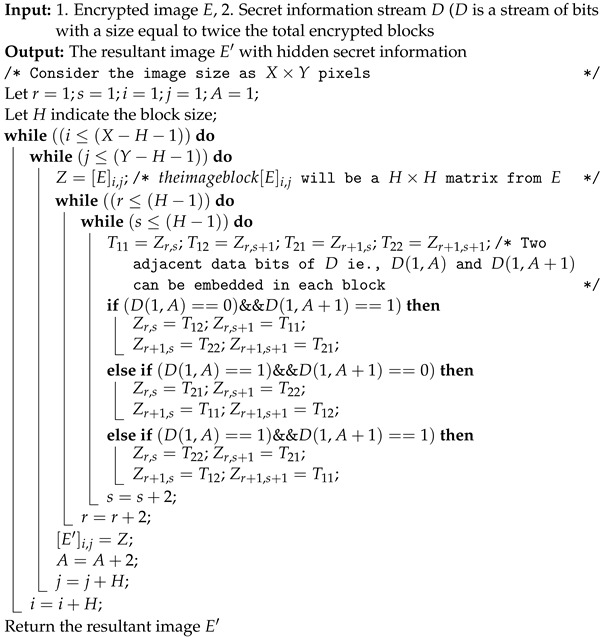


Let $d_{j}$ be the two bits that are embedded in the image block $E_{H\times H}^{i}$. Here,

(6) $$d_{j}=\left(X_{2j-1}X_{2j}\right),\;\;\;\;\mathrm{where}\;\;\;j=1,2,3,\ldots ,N.$$

Thus, the total embeddable secret information

(7) $$D=d_{1}∥d_{2}∥\ldots ∥d_{N}.$$

Furthermore,

(8) $$d_{j}\in \left\{00,01,10,11\right\},\;\;\;\;\mathrm{where}\;\;\;1\leq j\leq N.$$

In the proposed local pixel-swapping process, four different ways are formulated to address the embedding of four different bits such as 00,01,10, and 11. Let $Z_{i}$ be a sub-block of size 2×2 pixels from a block of 4×4 pixels. The pixels in $Z_{i}$ will undergo certain local pixel-swapping operations while embedding a two-bit sequence. The local pixel-swapping operations to embed a piece of 2-bit information in a 2×2-pixel block is as follows:

$$\mathrm{if}Z_{i}=\left[\begin{array}{cc} T_{11} & T_{12}\\ T_{21} & T_{22} \end{array}\right]\;\;\;\;\mathrm{then},$$

(9) $$Z_{i}^{\prime }=\left\{\begin{array}{cc} \left[\begin{array}{cc} T_{11} & T_{12}\\ T_{21} & T_{22} \end{array}\right] & \mathrm{if}\;d=00,\\ \left[\begin{array}{cc} T_{12} & T_{11}\\ T_{22} & T_{21} \end{array}\right] & \mathrm{if}\;d=01,\\ \left[\begin{array}{cc} T_{21} & T_{22}\\ T_{11} & T_{12} \end{array}\right] & \mathrm{if}\;d=10,\\ \left[\begin{array}{cc} T_{22} & T_{21}\\ T_{12} & T_{11} \end{array}\right] & \mathrm{if}\;d=11. \end{array}\right.$$

The above-mentioned technique is computed on each 2×2 block of $E_{H\times H}^{j}$ to embed a two-bit data $d_{j}$. Following the procedure, all the secret information gets embedded into the encrypted cover image producing a marked image $E^{\prime }$. The block size, H×H, is empirically decided. The marked image $E^{\prime }$ is transmitted to the receiver. We make a basic assumption that the decryption key *M* is already shared with the receiver.

### 3.2. Image Restoration Phase

The process of extracting the hidden secret information and the restoration of the cover image from the marked image $E^{\prime }$ is detailed in this section. The receiver uses Algorithm 2 to accomplish this phase. The image $E^{\prime }$ and the decryption key *M* make up the inputs to this phase. At first, we generate a pseudo-random matrix $M^{\prime }$ of size X×Y pixels using the key *M*, which will hold values in the range [0−255]. Further, the marked image is processed as independent blocks of fixed size H×H pixels, and each block is processed as independent 2×2-sized blocks, similar to the data-hiding phase.

The receiver employs the same local pixel-swapping process to recover the 2-bit information hidden in each block of the image $E^{\prime }$. Each block will be transformed into four different versions by using the four local pixel-swapping operations as referred to in Equation (9). Let us consider a marked block $V^{\prime }$ from $E^{\prime }$. One version of $V^{\prime }$ is generated by computing one type of local pixel-swapping operation on each 2×2-pixel block of $V^{\prime }$. Similarly, three other versions are also generated. If we consider $V^{\prime }$ as a 2×2-pixel block, then four different pixel arrangements, say $Z^{0},Z^{1},Z^{2}$, and $Z^{3}$, are generated as shown below. Let us assume

(10) $$Z=\left[\begin{array}{cc} T_{11} & T_{12}\\ T_{21} & T_{22} \end{array}\right].$$

Then, the four different pixel arrangements, $Z^{0},Z^{1},Z^{2},$ and $Z^{3}$ will be

(11) $$Z^{0}=\left[\begin{array}{cc} T_{11} & T_{12}\\ T_{21} & T_{22} \end{array}\right].$$

(12) $$Z^{1}=\left[\begin{array}{cc} T_{12} & T_{11}\\ T_{22} & T_{21} \end{array}\right].$$

(13) $$Z^{2}=\left[\begin{array}{cc} T_{21} & T_{22}\\ T_{11} & T_{12} \end{array}\right].$$

(14) $$Z^{3}=\left[\begin{array}{cc} T_{22} & T_{21}\\ T_{12} & T_{11} \end{array}\right].$$

**Algorithm 2:** Secret data extraction and image restoration.

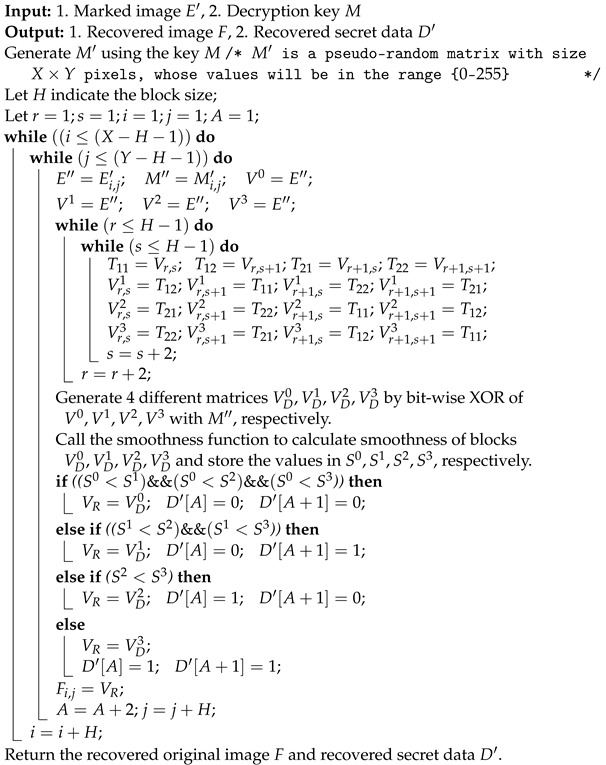



Applying Equation (11) to all the non-overlapping 2×2 blocks of $V^{\prime }$ will yield, say, $V^{0}$. Similarly, we obtain the remaining three image blocks of $V^{\prime }$, say, $V^{1},V^{2}$, and $V^{3}$, on applying Equations (12) through (14). We perform decryption through bit-wise XOR operation of the four different image blocks of $V^{\prime }$ and the corresponding block of pseudo-random matrix $M^{\prime }$; i.e., if the block $V^{\prime }$ is the *K*th block of $E^{\prime }$, we choose the corresponding *K*th block of $M^{\prime }$ to decrypt all the four image blocks {$V^{0},V^{1},V^{2}$, and $V^{3}$}. After decryption, we obtain new blocks $V_{D}^{0},V_{D}^{1},V_{D}^{2},$ and $V_{D}^{3}$.

(15) $$V_{D}^{0}=V^{0}⊕{M^{\prime }}^{K}.$$

(16) $$V_{D}^{1}=V^{1}⊕{M^{\prime }}^{K}.$$

(17) $$V_{D}^{2}=V^{2}⊕{M^{\prime }}^{K}.$$

(18) $$V_{D}^{3}=V^{3}⊕{M^{\prime }}^{K}.$$

The correlation between pixels in the decrypted blocks $\left\{V_{D}^{0},V_{D}^{1},V_{D}^{2},V_{D}^{3}\right\}$ paves the way for recovering the correct block. This is defined via a smoothness function. The algorithm for computing the multi-directional smoothness measure of a block is given in Algorithm 3. The input to Algorithm 3 is a decrypted image block $V_{D}^{i}$ of size H×H pixels, where 0≤ *i* <4. The computation of the multi-directional smoothness measure considers every overlapping 2×2 block of the decrypted block $V_{D}^{i}$. Consider *Q* as one of the 2×2 blocks from a decrypted block $V_{D}^{i}$; i.e., let

$$Q=\left[\begin{array}{cc} T_{11} & T_{12}\\ T_{21} & T_{22} \end{array}\right]\;\;then,$$

The smoothness measure SM of the 2×2 block *Q* is computed as follows:

(19) $$SM_{2\times 2}=\sum_{n=1}^{6}D_{n},$$

where

$$D_{1}=|T_{11}-T_{12}|.$$

$$D_{2}=|T_{21}-T_{22}|.$$

$$D_{3}=|T_{11}-T_{21}|.$$

$$D_{4}=|T_{12}-T_{22}|.$$

$$D_{5}=|T_{11}-T_{22}|.$$

$$D_{6}=|T_{12}-T_{21}|.$$

The smoothness measure of every overlapping 2×2 block of $V_{D}^{i}$ will be calculated. The sum of all these values gives the final smoothness measure. The smoothness measure is an indication of the strength of pixels correlation within a block. We expect a weak correlation among the pixels after encryption. Hence, the block $V_{D}^{i}$ with minimum smoothness value is the restored image block. Note that an incorrect block from $V_{D}^{i}$ will have a random pixel distribution, resulting in a higher smoothness value. The recovered block, through the smoothness measurement, is placed at the corresponding position of the restored image.

Consider $SM^{0},SM^{1},SM^{2},$ and $SM^{3}$ to be the smoothness measure computed from $V_{D}^{0},V_{D}^{1},V_{D}^{2}$, and $V_{D}^{3}$, respectively. If $SM^{m}$ gives the minimum measurement from $SM^{0},\;SM^{1},$ $SM^{2},\;SM^{3}$ where m∈{0,1,2,3} then the restored image block and the extracted hidden bits are determined as follows:

- Case 1: If m==0 then the 00 will be extracted message bits and $V_{D}^{0}$ will be the restored block.
- Case 2: If m==1 then the 01 will be extracted message bits and $V_{D}^{1}$ will be the restored block.
- Case 3: If m==2 then the 10 will be extracted message bits and $V_{D}^{2}$ will be the restored block.
- Case 4: If m==3 then the 11 will be extracted message bits and $V_{D}^{3}$ will be the restored block.

I.e., the correct block identified via the smoothness measurement calculation helps in recovering the hidden data in that block. It is obvious that the data hidden are 00 if the recovered block is $V_{D}^{0}$. This is because the decrypted block $V_{D}^{0}=V^{0}⊕{M^{\prime }}^{K}$ and $V^{0}$ is generated by applying Equation (11), which corresponds to the operation employed for embedding 00. This implies that 01 are the hidden data when the recovered block is $V_{D}^{1}$, 10 are the hidden data when the recovered block is $V_{D}^{2}$, and 11 are the hidden data when the recovered block is $V_{D}^{3}$. Likewise, all the marked image blocks are processed to obtain the original image without compromising the original quality of the cover image and recovering the hidden secret information parallelly. Algorithm 2 returns the restored image *F* and the extracted message *D*. An example of the proposed MPSA scheme is given in the next section of this manuscript for better comprehension.
**Algorithm 3:** Computing multi-directional smoothness measure.
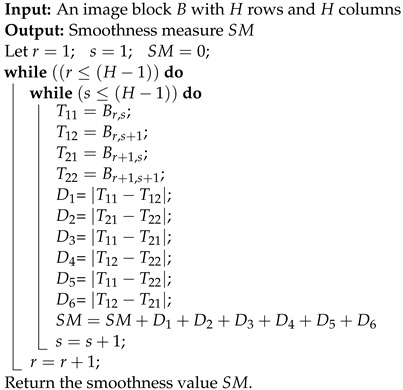


## 4. Illustrative Example

**Figure 4:**
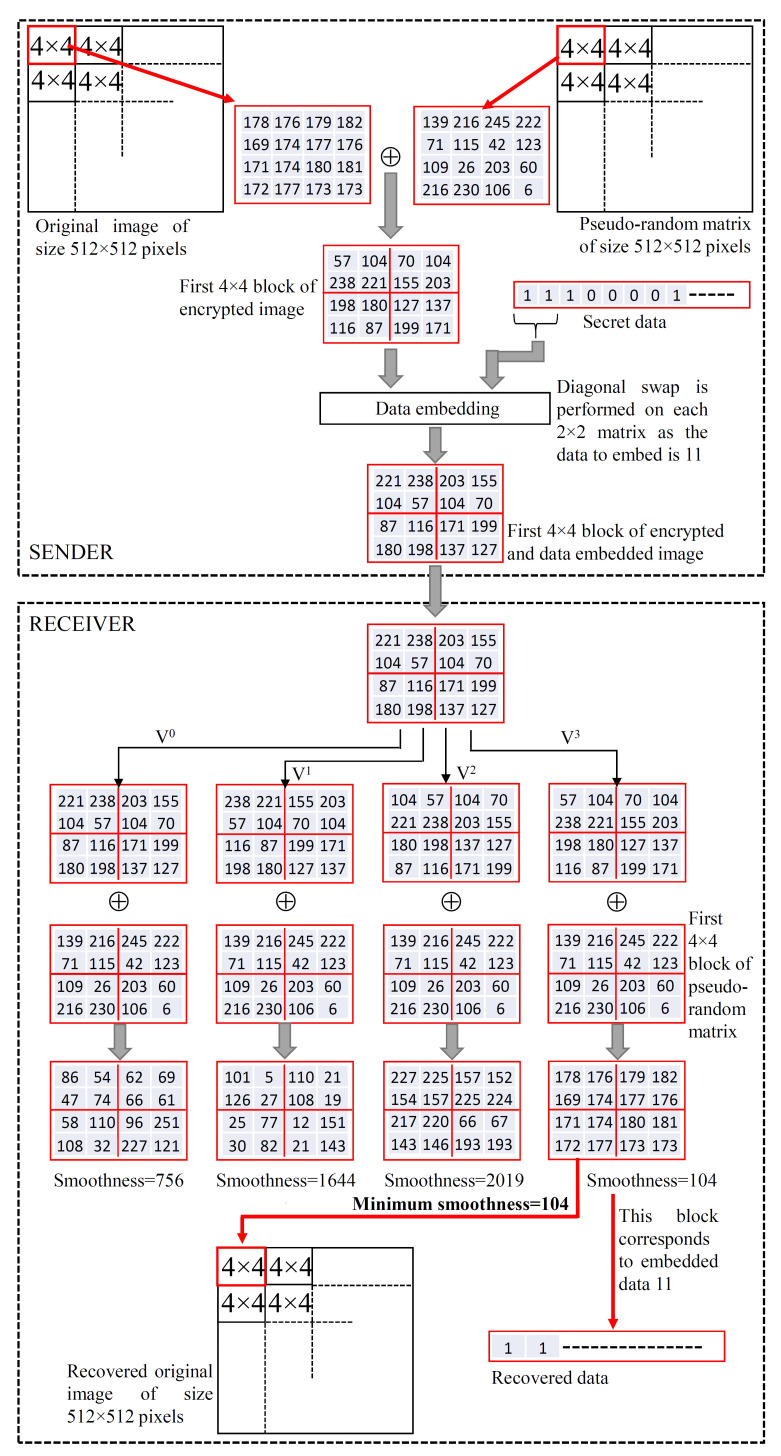
Illustration of the proposed scheme.

The sequence of operations performed to hide the data and to restore the image is schematically shown in Figure 4. An original image of size 512×512 pixels is considered for illustration. An encryption key is used to generate a similar-sized pseudo-random matrix. The image and the pseudo-random matrix are bit-wise XORed to obtain an encrypted image. The illustration shown in Figure 4 considers the processing of the image as independent 4×4 pixel blocks. A series of binary numbers form the secret information, out of which 2 bits of information are embedded on each independent 4×4 block. As shown in the example, the 2-bit data which we need to hide in the selected block are 11. To embed 11, we perform a local diagonal swapping of the pixels (refer to Equation (9)) on each non-overlapping 2×2 pixel block of the currently selected 4×4 block. This makes the initially encrypted block embedded with secret data 11. Now the information embedded 4×4 block is placed at the corresponding position of the marked image $E^{\prime }$. The marked image $E^{\prime }$ is transmitted to the receiver.

The receiver also processes the received image $E^{\prime }$ as independent 4×4 pixel blocks. In the illustrative example, we considered the same 4×4 data-embedded block to justify how we restore the cover image and extract the exact hidden data. The receiver generates four different versions of the data-embedded 4×4 block, which are $V^{0},V^{1},V^{2},$ and $V^{3}$. Bit-wise XOR over these blocks with the corresponding block from the pseudo-random matrix performs the decryption operation. The pseudo-random matrix is created using the decryption key. We compute the smoothness measure of all these four decrypted blocks to recover the correct block (refer to Algorithm 3). The restored block is the one with the lowest smoothness measure. Say, if the decrypted block recovered is computed from version $V^{P}$, then the hidden data extracted are *P* where P∈0,1,2,3. As shown in the example, the fourth decrypted version of the block is the block with the lowest measure. Hence, this block is the correct block recovered. This version of the decrypted block is computed from $V^{3}$, which corresponds to the data 11. In a similar fashion, all the remaining blocks of the marked image are processed to restore the original image and the complete hidden message.

## 5. Experimental Study

The RDH scheme can secure both the cover image and the secret information. To validate the proposed MPSA scheme, we implemented the proposed algorithms using Matlab R2019a. The standard image dataset available from the University of Southern California, which is popularly known as the USC-SIPI images [39], and the BOWS-2 [40] database are used for experimentation. All the images were prepared into a 512×512 pixel image, before being given as input to the proposed MPSA system. The secret information used for this study is a series of pseudo-random binary bits generated using a pseudo-random generator, and the length of the bit sequence is fine-tuned based on the number of blocks used during data hiding. The following parameters are considered for validation of the newly introduced scheme:

1. Embedding rate (ER)
2. Bit error rate (BER)
3. Peak signal-to-noise ratio (PSNR)
4. Structural similarity index (SSIM)
5. Entropy
6. Histogram analysis
7. Image recovery analysis
8. Computation time

In the next sub-sections, we discuss all the above-mentioned results in detail.

### 5.1. Inspection on the Embedding Rate (ER) and Data Recovery

Embedding capacity, payload capacity, or embedding rate are different terms used by researchers working in the domain of data hiding, which eventually points to the same context. It refers to the quantity of data that one can hide on a cover medium. This has been an ongoing research activity to improve the embedding rate by introducing a new algorithm or by modifying the existing schemes. Embedded rate (ER) can be expressed as follows:

(20) $$ER=\left(\frac{N_{B}}{T_{P}}\right).$$

where $N_{B}$ is the length of data embedded in the cover image and $T_{P}$ is the total pixels present in the image.

Bit error rate (BER) is also an important parameter in any RDH approach. This parameter is an implication of how efficiently the RDH algorithm retrieves the embedded secret information. BER refers to the bulk of data recovered incorrectly, considering the quantity of data that was hidden in the cover medium. The best schemes should result in a BER value of 0.

**Figure 5:**
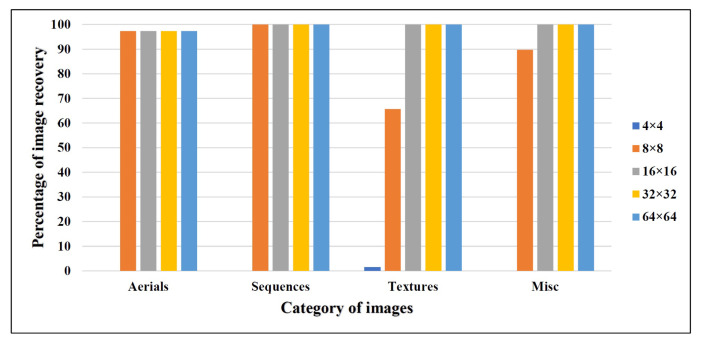
Percentage of image recovery on USC-SIPI dataset.

**Figure 6:**
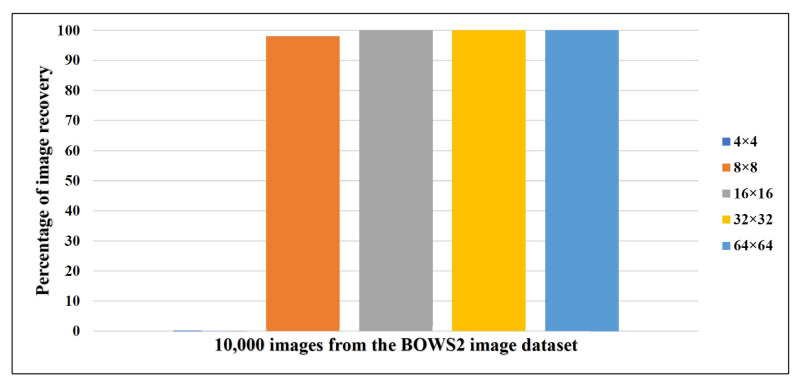
Percentage of image recovery on BOWS-2 dataset.

The ER that is achievable from the MPSA scheme purely relies on the processed block size. A block size of H×H pixels results in an ER of $\frac{1}{H^{2}}$ bits per pixel (bpp). The use of small block sizes will help to obtain more partitions in the image and which in turn leads to higher embedding. However, the use of appropriate block size affects the BER, which is critical in restoring the correct image and hence extracting the hidden data. BER is analyzed with varying block sizes. The percentage of successful image recovery while trying with various block sizes on all four categories of images of the USC-SIPI dataset and BOWS-2 dataset are graphically shown in Figure 5 and Figure 6, respectively.

In addition to this, we analyzed the BER from well-known images such as airplane, baboon, boat, and peppers to showcase the results of the experiment. Table 1 shows the effective embedding rate and BER. Table 1 clearly implies that the MPSA scheme restores the correct cover image and the embedded secret information completely in the case of 8×8,16×16,32×32, and 64×64 block processing, except for the case of 8×8 block processing on the baboon.

The results claim that the three well-known images, *airplane, boat,* and *peppers*, are restored without any kind of error with an 8×8-pixel block size. This brings in an ER of $\frac{2}{8^{2}}=0.03125$ bpp. However, the *baboon* image was restored completely only when the block size was 16×16 pixels. Hence, the embedding rate of the *baboon* image as per the newly introduced scheme is $\frac{2}{16^{2}}=0.00781$ bpp. Compared to other images, the *baboon* image has a highly textured property that leads to more errors when processed with low block sizes. This led to a lower embedding rate of the baboon image. It is significant to note that a block of size 4×4 pixels contains fewer pixels compared to larger block sizes. Hence, most of the pixels in a smaller block with edges or corners in it will not have a strong correlation. This effect will be lower with higher block size pixels. Our scheme recovers the block by computing the smoothness measure that is related to the correlation of the pixels. The edges or corners in a 4×4-pixel block adversely affect the pixel correlation, reflecting a higher smoothness value. In this manuscript, first, we recover the correct block via the smoothness measurement process, and data extraction depends on this recovered block. Hence, when the correct block is not recovered through the smoothness measurement process, we fail to extract the correct hidden information. This justifies a non-zero BER with lesser block sizes, as mentioned in Table 1.

### 5.2. Analysis of Peak Signal-to-Noise Ratio (PSNR) and Structural Similarity Index (SSIM)

PSNR is a factor of the maximum possible value of a pixel to the error that distorts the original quality of the image. It is expressed as follows:

(21) $$PSNR=10\log_{10}\left(\frac{I_{max}^{2}}{MSE}\right)\;\;\mathrm{dB},$$

where $I_{max}$ indicates the maximum possible value of an image pixel and MSE is the mean squared error, defined as follows:

(22) $$MSE=\left(\frac{1}{XY}\sum_{a=1}^{X}\sum_{b=1}^{Y}{\left|I\left(a,b\right)-R\left(a,b\right)\right|}^{2}\right),$$

where the reference image is *I* and the restored image is *R*. The MPSA scheme is expected to restore the correct cover image losslessly, and hence MSE = 0. This implies that PSNR should give *∞* for a good approach.

The SSIM gives the structural similarity index, another parameter for effective quality estimation of the restored image. Here, the structural quality degradation of the restored image gets quantified based on the luminance masking and contrast masking factors. SSIM should be 1 for the correctly recovered original image. The results of PSNR and SSIM values are also tabulated in Table 2.

The PSNR values returned from the MPSA scheme on well-known images, along with the SSIM outputs, are indicated in Table 2. All three images, except *baboon*, were restored without any loss of information. This yields a PSNR *∞* and SSIM measure of 1 with a block size of 8×8 pixels and higher. As mentioned in the previous section, our scheme recovers the correct block via the smoothness measurement, which is a reflection of the correlation between the pixels in the block. A smaller block with edges or corners does not give significant scope for a good smoothness measure. Hence, perfect recovery of a smaller block is difficult, as evident from the PSNR and SSIM values for 4×4-pixel block sizes in Table 2.

### 5.3. Analysis of Efficiency of Image Encryption

The encryption algorithm’s efficiency can be analyzed from (a) the entropy of the encrypted image and (b) the histogram of the encrypted image. Entropy estimates the level of randomness of an image pixel. It characterizes the texture of the image. The entropy is defined as follows:

(23) $$E\left(I\left(Y\right)\right)=\sum_{l=0}^{Y-1}P\left(L\right)\log_{2}\frac{1}{P(L)}\;,$$

where *I* is the image, *Y* indicates the peak gray level (256 for a grayscale image), and P(L) indicates the probability of a pixel with intensity value *L*. If the encrypted image is highly random, then the entropy measure should be near 8 for a grayscale image.

The value of entropy measured over the original cover image, the image acquired through encrypting the cover image, and the image acquired from hiding a given sequence of secret data are given in Table 3. It is worth mentioning that the pixels of the encrypted image and the image acquired from the data-embedding process are both the same (only the position of the pixels is changed), and hence the entropy measurement of both images is also the same. This confirms that the security level of the marked image is as strong as the encrypted image, further substantiating the title of the paper. It is apparent from Table 3 that the encrypted image entropy remains the same after the data-hiding process.

**Figure 7:**
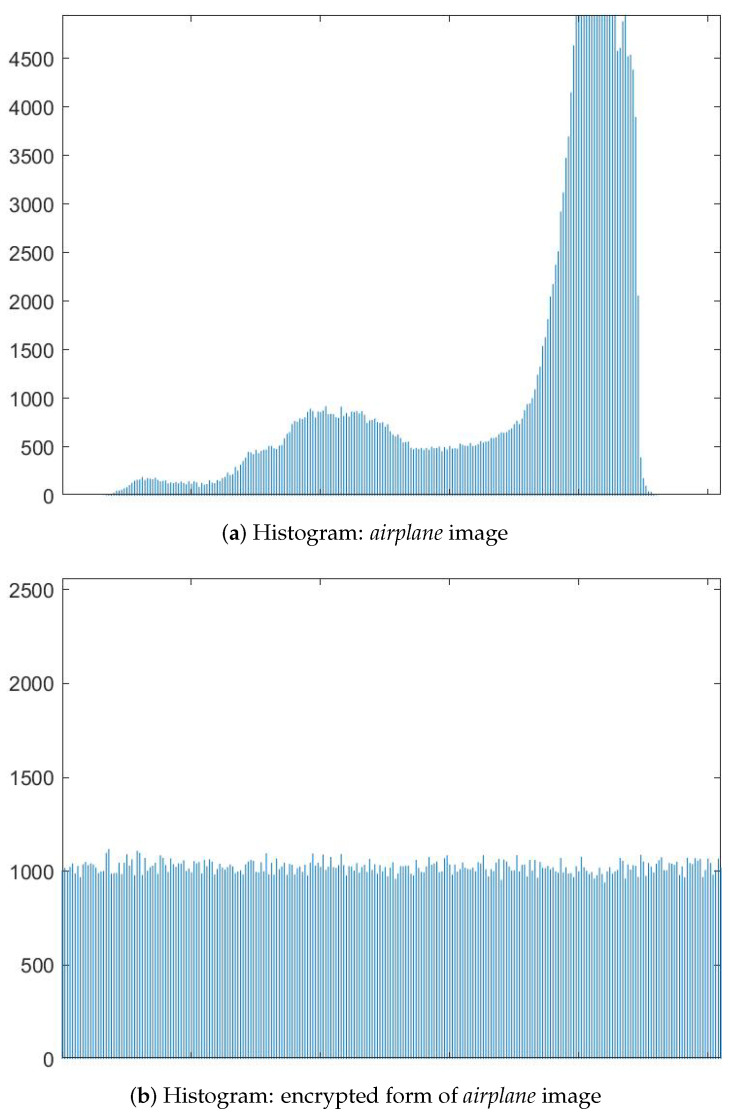

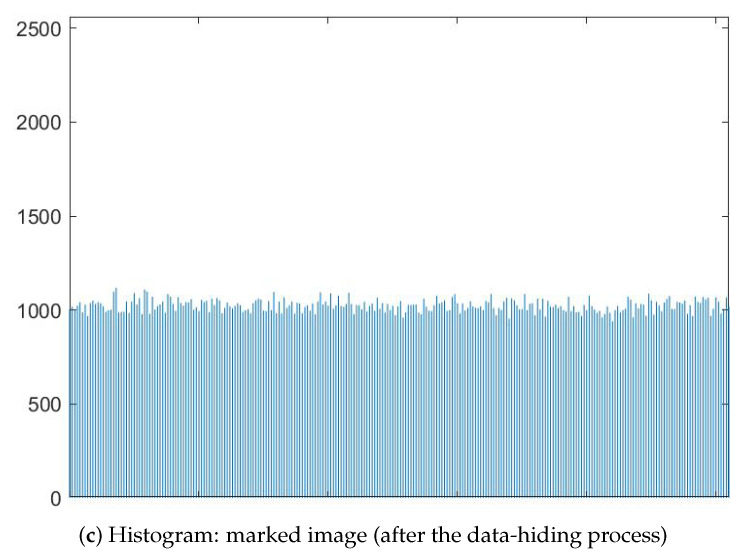
Histogram analysis on *airplane* image.

The histogram of an image indicates how the image pixels are distributed. Any kind of changes that occur during data hiding will alter the original image’s pixel distribution. A uniform distribution of pixel values is anticipated from an encrypted image. In such a case, the encrypted image histogram will look flat. Hence, changing encrypted image pixel values during the data-hiding process can lead to a different histogram. However, as we discussed earlier, the proposed MPSA scheme makes no attempt to change the encrypted image histogram. The histogram acquired over the original *airplane* image, over the image acquired through encrypting the image, and over the image acquired from hiding a given sequence of secret data is shown in Figure 7. As encrypted image pixel intensities are uniformly distributed, the histogram of the encrypted image looks flat in Figure 7. Furthermore, the marked image histogram is the same as the encrypted image histogram, implying that the image encryption efficiency is favored through the proposed data-hiding process of the MPSA scheme.

### 5.4. Justification for Image Recovery

**Figure 8:**
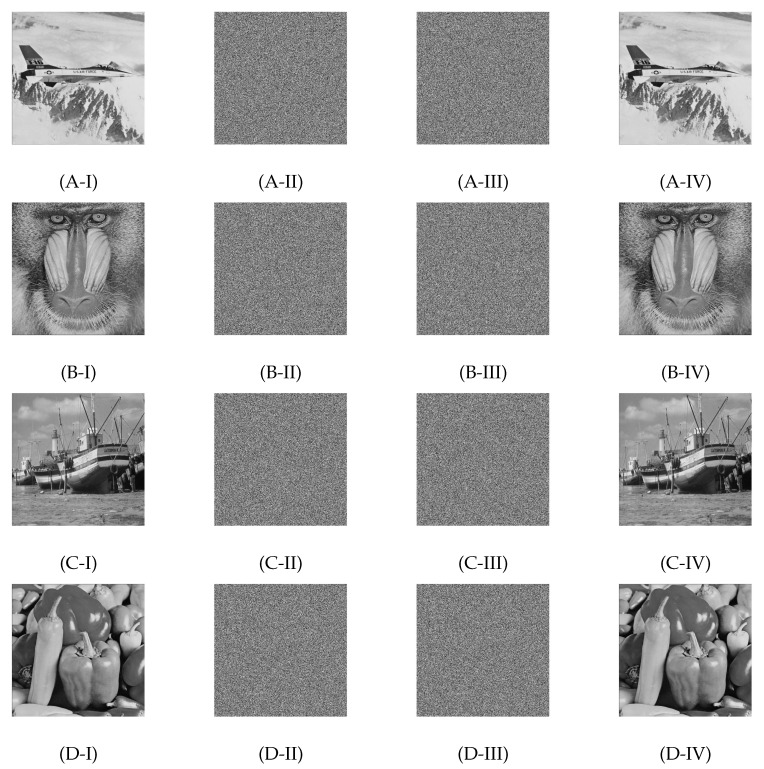
Sample results acquired in the course of experiments. Original images: (**A-I**,**B-I**,**C-I**,**D-I**); corresponding encrypted images: (**A-II**,**B-II**,**C-II**,**D-II**); corresponding marked images: (**A-III**,**B-III**,**C-III**,**D-III**); corresponding restored images: (**A-IV**,**B-IV**,**C-IV**,**D-IV**).

The images in the USC-SIPI image dataset are of four different categories: *Misc., Aerials, Sequences*, and *Textures*. We considered all 39 *Misc.* images, 38 *Aerials* images, 69 *Sequences* images, and 64 *Textures* images of the USC-SIPI image dataset and 10,000 images from the BOWS-2 dataset for experiments. The image recovery statistics are given in Figure 5 and Figure 6. An image is considered to be successfully recovered when there is no loss of information. It is observed from Figure 5 and Figure 6 that most of the images were successfully recovered when the processed block size was set to 8×8 pixels. It should be noted that the images that were not restored properly with a block size of 8×8 pixels were completely recovered with a block size of 16×16 pixels. Some of the sample results acquired in the course of experiments are given in Figure 8.

The success of the proposed MPSA scheme lies in the smoothness measure analysis. We believe that the adjacent pixels in a natural image are highly correlated. This property was exploited in the proposed scheme during the image restoration phase. Algorithm 3 analyzes the pixel differences in all possible directions, such as horizontal, vertical, and diagonal. Hence, a correctly recovered block is expected to give a low measure since the adjacent pixel intensities do not vary much. However, pixels in the highly textured area and with the presence of edges can certainly produce a high difference.

**Figure 9:**
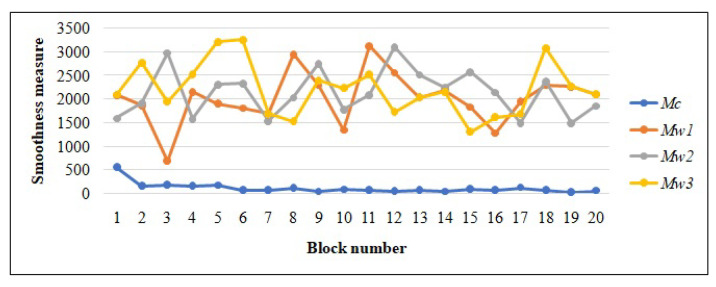
The difference in smoothness measure between the correctly restored block and the other three corresponding incorrect blocks during the image restoration process.

We selected 20 random blocks during the image recovery process of the *airplane* image to analyze the computed smoothness measure. The block size used for the analysis is of size 4×4 pixels. Note that during the image restoration and data extraction phase, we try to discover the correct block from four decrypted varieties of the image block. The correctly restored block is identified based on the smoothness measure. The block which will give the lowest smoothness measure is considered the correctly recovered block. Figure 9 shows the smoothness measure obtained from the four decrypted varieties of all 20 image blocks. In Figure 9, the smoothness measure of each correctly restored block is indicated by Mc, which is too small in comparison with the measure from corresponding incorrect blocks Mw1,Mw2, and Mw3.

### 5.5. Comparative Study

A comparison of the proposed MPSA scheme with a few well-known existing RDH schemes, reported in [21,22,27,41,42,43,44,45,46,47,48], is discussed in this section. Table 4 gives a comparative study of the embedding rate considering well-known images such as *airplane, baboon, boat*, and *peppers*. All three images, except the *baboon* image, are recovered correctly when 8×8 pixels is set as the processed block size, leading to an embedding rate of 0.03125 bpp. *Baboon* is a highly textured image that was restored successfully with 16×16 pixels or more as the processed block size. Hence, the highest embedding rate on *baboon* was reduced to 0.0078 bpp. Still, it gives an acceptable rate, superior to many other existing schemes.

The results listed in Table 4 highlight a *better* embedding efficiency of the proposed MPSA scheme in comparison to other well-known existing RDH schemes for encrypted images. It should be noted that the comparison of the schemes is not just based on the embedding rate, but all the compared schemes provide a complete recovery with a PSNR of *∞* and SSIM of 1. It is noteworthy to mention that none of the schemes other than the proposed scheme retains the entropy of the encrypted image and, similarly, the histogram of the encrypted image despite embedding the secret information.

### 5.6. Real-Time Execution of the Proposed MPSA Scheme

**Figure 10:**
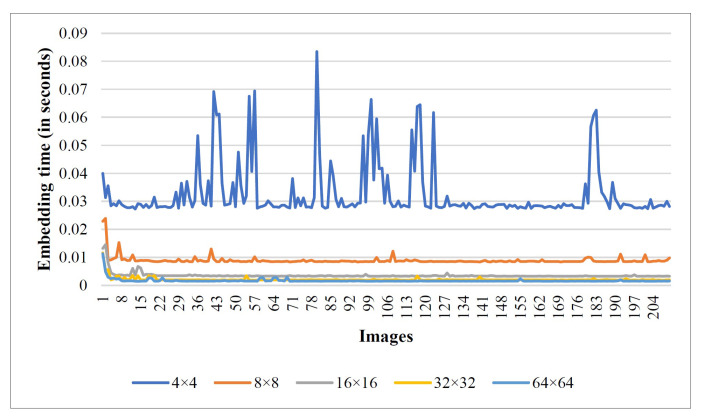
Execution time taken by USC-SIPI dataset images during the data-embedding phase.

**Figure 11:**
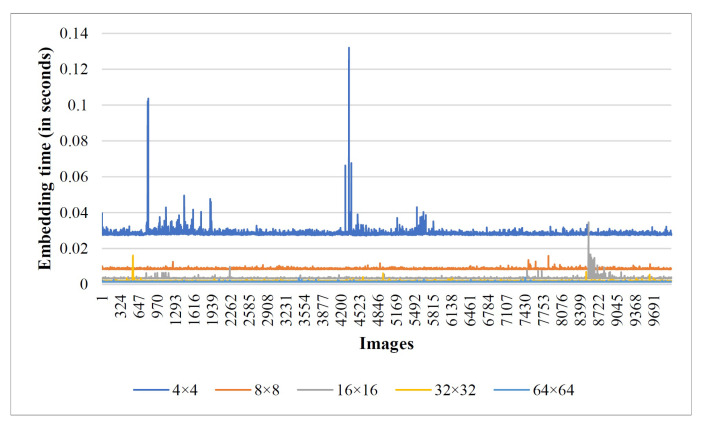
Execution time taken by different images of the BOWS-2 dataset during the data-embedding phase.

**Figure 12:**
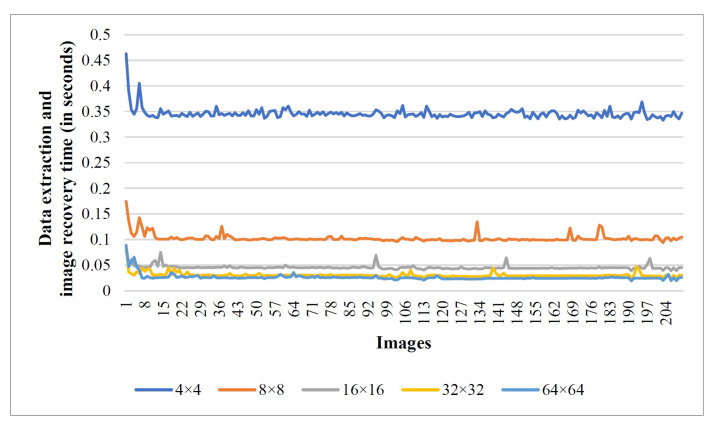
Execution time taken by USC-SIPI dataset images during the data recovery and image restoration phase.

**Figure 13:**
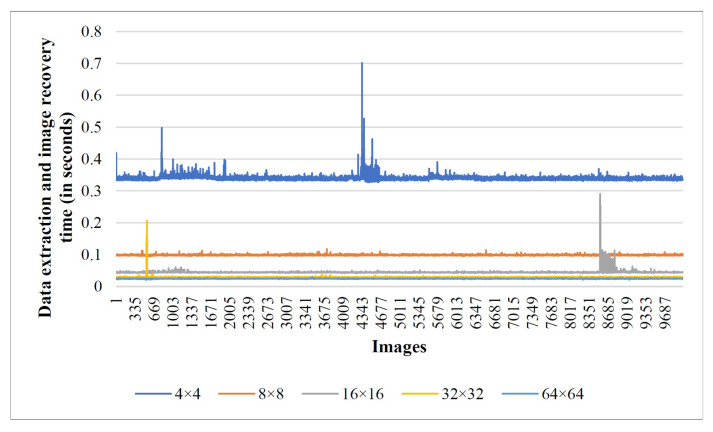
Execution time taken by BOWS-2 dataset images during the data recovery and image restoration phase.

The system configuration employed for the experiments includes a x64-based Intel(R) Core(TM) i5-9300H CPU @ 2.40GHz with 8GB RAM. In a real-time scenario, the efficiency of an algorithm predominantly rests on time complexity. Here, we disclose the execution time taken by the proposed MPSA RDH scheme that supports its real-time applicability. The graph given in Figure 10 and Figure 11 reveals the experimental time taken during secret data hiding in the encrypted image through the proposed MPSA scheme on the USC-SIPI and BOWS-2 image datasets, respectively. All images are standardized to 512×512 size pixels before computing the execution time. Figure 12 and Figure 13 show the time taken by each image while extracting the hidden information and recovering the original cover image. Note that we used the USC-SIPI image dataset, which contains 210 images, and the BOWS-2 dataset, which contains 10,000 images in total, for the experiments. It should be noted that each line in the graph indicates the scenario of processing the image at a particular block size. Based on the experimental study, it is inferred that the computation time taken during the embedding and recovery phase is high while processing the image as 4×4 size pixels, and it decreases with larger block size processing of the image.

The average execution time incurred by images under various classes of the USC-SIPI dataset while processing as 8×8-sized pixels is tabulated in Table 5. As we can observe, the worst-case average embedding time is 0.0106 s, and the worst-case average time during the data recovery and image restoration phase is 0.1090 s, while the average embedding time considering 10,000 images from the BOWS-2 dataset with 8×8 block processing is 0.0086 s, and the average restoration time is 0.0986 s. This proves the real-time applicability of the proposed MPSA scheme. Moreover, all the RDH schemes mentioned in Table 4, including the proposed scheme, have a theoretical time complexity of O(*K*), where K=M.N. Here, *M* is the number of rows in the processed image, and *N* is the number of columns in the processed image.

## 6. Conclusions

This paper proposes a computationally efficient and novel entropy and histogram retained MPSA RDH scheme. The majority of the existing RDH schemes modify the encrypted cover image pixels to hide the secret data, raising security concerns over the operated encryption algorithm. The entropy of the encrypted image and the histogram of the encrypted image are two useful parameters that quantify the encryption algorithm’s efficiency. Our proposed MPSA scheme is completely reversible and is suitable for any encryption algorithm. The proposed data-hiding process does not change the encrypted cover image pixel intensities, maintaining the same entropy and the same histogram of the encrypted cover image. Thus, the encryption efficiency of the encryption algorithm is not compromised. Moreover, the data-embedding process is independent of the structure or any statistical measures of the cover image, achieving a constant embedding rate on all images. The proposed MPSA allows an embedding of 0.03125 bits per pixel for most of the USC-SIPI images and the BOWS-2 image dataset. In the future, we would like to improve the entropy retained RDH scheme with enhanced embedding capacity without compromising image recoverability.

## Figures and Tables

**Table 1:** Embedding rate and BER obtained while using different block sizes.

Name of the Image	Block Length	Embedding Rate	BER
Airplane	4×4	0.12500	0.00617
	8×8	0.03125	0
	16×16	0.00781	0
	32×32	0.00195	0
	64×64	0.00049	0
Baboon	4×4	0.12500	0.01849
	8×8	0.03125	0.00024
	16×16	0.00781	0
	32×32	0.00195	0
	64×64	0.00049	0
Boat	4×4	0.12500	0.00604
	8×8	0.03125	0
	16×16	0.00781	0
	32×32	0.00195	0
	64×64	0.00049	0
Peppers	4×4	0.12500	0.00275
	8×8	0.03125	0
	16×16	0.00781	0
	32×32	0.00195	0
	64×64	0.00049	0

**Table 2:** Analysis of PSNR (in dB) and SSIM.

Image Used	Block Length	PSNR	SSIM
Airplane	4×4	27.66	0.97
	8×8	*∞*	1
	16×16	*∞*	1
	32×32	*∞*	1
	64×64	*∞*	1
Baboon	4×4	24.02	0.94
	8×8	43.34	0.99
	16×16	*∞*	1
	32×32	*∞*	1
	64×64	*∞*	1
Boat	4×4	28.58	0.97
	8×8	*∞*	1
	16×16	*∞*	1
	32×32	*∞*	1
	64×64	*∞*	1
Peppers	4×4	31.09	0.99
	8×8	*∞*	1
	16×16	*∞*	1
	32×32	*∞*	1
	64×64	*∞*	1

**Table 3:** Analysis of entropy measure.

Image Used	Block Length	Entropy (Original Image)	Entropy (After the Encryption Process)	Entropy (After the Data-Embedding Process)
Airplane	4×4	6.7025	7.9993	7.9993
	8×8	6.7025	7.9993	7.9993
	16×16	6.7025	7.9993	7.9993
	32×32	6.7025	7.9993	7.9993
	64×64	6.7025	7.9993	7.9993
Baboon	4×4	7.3583	7.9994	7.9994
	8×8	7.3583	7.9994	7.9994
	16×16	7.3583	7.9994	7.9994
	32×32	7.3583	7.9994	7.9994
	64×64	7.3583	7.9994	7.9994
Boat	4×4	7.1914	7.9993	7.9993
	8×8	7.1914	7.9993	7.9993
	16×16	7.1914	7.9993	7.9993
	32×32	7.1914	7.9993	7.9993
	64×64	7.1914	7.9993	7.9993
Peppers	4×4	7.5937	7.9992	7.9992
	8×8	7.5937	7.9992	7.9992
	16×16	7.5937	7.9992	7.9992
	32×32	7.5937	7.9992	7.9992
	64×64	7.5937	7.9992	7.9992

**Table 4:** Distinguishing embedding rate of different RDH schemes for the well-known images.

Scheme	Airplane	Baboon	Boat	Peppers	Entropy Retained	Histogram Retained
LSB replacement based RDH [21]	0.0039	0.0009	0.0039	0.0015	✗	✗
Side match approach based RDH [22]	0.0039	0.0009	0.0039	0.0039	✗	✗
LSB compression based RDH [41]	0.0300	0.0100	0.0300	0.0300	✗	✗
Public key cryptosystem based RDH [42]	0.0020	0.0020	0.0020	0.0020	✗	✗
Absolute mean difference based RDH [27]	0.0020	0.0008	0.0049	0.0039	✗	✗
Difference expansion + Public key cryptosystem [43]	0.0040	0.0040	0.0040	0.0040	✗	✗
Histogram compression based RDH [44]	0.0080	0.0080	0.0080	0.0080	✗	✗
Histogram shifting + Public key cryptosystem [45]	0.0080	0.0080	0.0080	0.0080	✗	✗
Mean value based RDH [46]	0.0039	0.0039	0.0039	0.0039	✗	✗
Joint RDH + Symmetric cryptosystem [47]	0.0048	0.0011	0.0072	0.0072	✗	✗
Pseudo-random pixel mapping + Weighted mesh graph RDH [48]	0.0313	0.0313	0.0313	0.0313	✗	✗
Proposed MPSA RDH	0.0313	0.0078	0.0313	0.0313	✔	✔

**Table 5:** Table showing the computed average execution time.

Image Database	Image Category	Average Data-Embedding Time (s)	Average Data Recovery and Image Restoration Time (s)
USC-SIPI	Aerials	0.0096	0.1069
	Sequences	0.0104	0.1079
	Textures	0.0089	0.1027
	Misc	0.0106	0.1090
BOWS-2	-	0.0086	0.0986

## Data Availability

The datasets generated during and/or analyzed during the current study are available in the following repositories: 1. USC-SIPI: https://sipi.usc.edu/database/ (accessed on 20 March 2023). 2. BOWS-2: http://bows2.ec-lille.fr/ (accessed on 20 March 2023).
